# A phase 2 randomised study of veliparib plus FOLFIRI±bevacizumab versus placebo plus FOLFIRI±bevacizumab in metastatic colorectal cancer

**DOI:** 10.1038/s41416-018-0343-z

**Published:** 2018-12-11

**Authors:** Vera Gorbunova, J. Thaddeus Beck, Ralf-Dieter Hofheinz, Pilar Garcia-Alfonso, Marina Nechaeva, Antonio Cubillo Gracian, Laszlo Mangel, Elena Elez Fernandez, Dustin A. Deming, Ramesh K. Ramanathan, Alison H. Torres, Danielle Sullivan, Yan Luo, Jordan D. Berlin

**Affiliations:** 1 N.N. Blokhin Russian Cancer Research Center, Moscow, Russia; 2 Highlands Oncology, Rogers/Fayetteville, AR USA; 3 Interdisciplinary Tumor Center, University Hospital Mannheim, University of Heidelberg, Heidelberg, Germany; 4 Hospital General Universitario Gregorio Marañón, Madrid, Spain; 5 Arkhangelsk Clinical Oncology Center, Arkhangelsk, Russia; 6 Centro Integral Oncológico Clara Campal Hospital Universitario Madrid Sanchinarro, Madrid, Spain, and Departamento de Ciencias Médicas Clínicas, Universidad CEU San Pablo, Madrid, Spain; 7 Pecsi Tudomanyegyetem Klinikai Kozpont, Onkoterapias Intezet, Pécs, Hungary; 8 Vall d’Hebron University Hospital, Barcelona, Spain; 9 University of Wisconsin, Madison, WI USA; 10 Mayo Clinic, Scottsdale, AZ USA; 11 AbbVie Inc., North Chicago, IL USA; 12 Vanderbilt-Ingram Cancer Center, Nashville, TN USA

**Keywords:** Colorectal cancer, Phase II trials, Chemotherapy, Colorectal cancer

## Abstract

**Background:**

Metastatic colorectal cancer (mCRC) has low survival rates. We assessed if addition of veliparib, concurrent to FOLFIRI, improves survival in patients with previously untreated mCRC.

**Methods:**

This study compared veliparib (200 mg BID for 7 days of each 14-day cycle) to placebo, each with FOLFIRI. Bevacizumab was allowed in both arms. The primary endpoint was progression-free survival (PFS).

**Results:**

Patients were randomised to receive veliparib (*n* = 65) or placebo (*n* = 65) in combination with FOLFIRI. Median PFS was 12 vs 11 months (veliparib vs placebo) [HR = 0.94 (95% CI: 0.60, 1.48)]. Median OS was 25 vs 27 months [HR = 1.26 (95% CI: 0.74, 2.16)]. Response rate was 57% vs 62%. Median DOR was 11 vs 9 months [HR = 0.73 (95% CI: 0.38, 1.40)]. AEs with significantly higher frequency (*p* < 0.05) in the veliparib group were anaemia (39% vs 19%, *p* = 0.019) and neutropenia (66% vs 37%, *p* = 0.001) for common AEs (≥20%); neutropenia (59% vs 22%, *p* < 0.001) for common Grade 3/4 AEs (≥5%); none in serious AEs. Haematopoietic cytopenias were more common with veliparib (79% vs 52%, *p* = 0.003). Fourteen percent of patients on veliparib and 15% on placebo discontinued treatment due to AEs.

**Conclusion:**

Veliparib added to FOLFIRI ± bevacizumab demonstrated similar efficacy as FOLFIRI ± bevacizumab in frontline mCRC patients. No unexpected safety concerns occurred.

## Introduction

Colorectal cancer is the third most common cancer in men and the second most common cancer in women.^1^ Metastatic colorectal cancer is the second leading cause of cancer death in the United States^2^, and the 5-year survival rate for advanced colorectal cancer remains at only ~11%.^3^ The majority of patients faced with a diagnosis of metastatic colorectal cancer have either extensive liver disease or disease outside the liver, limiting their treatment options to chemotherapy.^4^ Cancer develops drug resistance over the course of treatment, in part, due to increased levels of DNA repair.^5,6^ Current frontline therapy includes a combination of irinotecan/5-fluorouracil/leucovorin (FOLFIRI) with or without bevacizumab.^7–14^ While the introduction of each of these regimens has resulted in incremental increases in patient survival,^15^ survival rates for patients with metastatic colorectal cancer remain low.

Veliparib is a potent, competitive poly (ADP-ribose) polymerase (PARP)-1/2 inhibitor that enhances the activity of topoisomerase I inhibitors, such as irinotecan, in preclinical models.^16–21^ Veliparib was shown to synergise irinotecan in killing of colon cancer cells in tissue culture.^22^ Therefore, patients receiving cytotoxic chemotherapy including irinotecan may benefit from the addition of veliparib to their treatment. A phase 1, open-label dose escalation study (NCT01123876) evaluated the safety and tolerability of veliparib in combination with modified bimonthly FOLFIRI^23^ demonstrated acceptable safety profile and preliminary antitumour activity of veliparib plus FOLFIRI. The phase 1 results support further evaluation of this combination in a phase 2 trial.

## Patients and methods

### Patients

Eligible patients were 18 years or older, with histologically or cytologically confirmed metastatic adenocarcinoma of the colon or rectum and had not received prior chemotherapy for their metastatic colorectal cancer. Patients had at least one unresectable lesion on a computed tomography scan that was measurable by Response Evaluation Criteria in Solid Tumours (RECIST), version 1.1, and had an Eastern Cooperative Oncology Group (ECOG) performance status of 0 or 1 and adequate haematologic, renal and hepatic function. Patients receiving bevacizumab also had blood pressure well controlled (<160/90 mmHg) on a stable regimen of anti-hypertensive therapy for at least 2 weeks.

Patients were not eligible if they had prior anti-cancer treatment for metastatic colorectal cancer, prior PARP inhibitors, or adjuvant/neoadjuvant chemotherapy within 1 year prior to Cycle 1 Day −2, or major surgery or prior radiotherapy within 1 month prior to Cycle 1 Day −2, or prior radiation to >25% of bone marrow. Patients were also excluded if they had known Gilbert’s Syndrome, bowel disorders with diarrhoea of more than three times daily, or need of parenteral nutrition, or bowl obstruction/perforation, any clinically significant and uncontrolled medical condition(s), were pregnant or lactating, or were at an unacceptable high risk for toxicities. Patients receiving bevacizumab in this trial were not eligible if they had prior treatment with bevacizumab, known central nervous system metastases, significant history of bleeding events or gastrointestinal perforation, serious or non-healing wound, ulcer, or bone fracture, or history of venous or arterial thromboembolism within 2 months of enrolment.

This study was conducted in accordance with the protocol, International Conference on Harmonisation Good Clinical Practice guidelines, applicable regulations and guidelines governing clinical study conduct and the ethical principles that have their origin in the Declaration of Helsinki. The study protocol was independently approved by the ethics committees of each participating centre. All patients provided written informed consent before participation in the trial.

### Study design and treatment

This randomised, phase 2 study was conducted at multiple sites globally (North America, Europe and Australia), and is registered at Clinicaltrials.gov, number NCT02305758. Patients were randomised to one of two treatment groups: veliparib plus FOLFIRI with or without bevacizumab, or placebo plus FOLFIRI with or without bevacizumab. Patients were stratified by the planned use of bevacizumab and world region, and randomisation was conducted to ensure at least 60 patients enrolled to both the planned and unplanned bevacizumab groups, for a planned sample size of ~120 patients. The primary objective of the study was to assess whether the addition of oral veliparib to FOLFIRI improves progression-free survival (PFS) in patients with previously untreated mCRC.

Patients were randomised and dosed with veliparib/placebo on Cycle 1 Day −2. Sample size was chosen by a minimum of 70 PFS events to provide adequate precision in the hazard ratio estimate. Assuming median PFS (mPFS) time of 9.4 months in the FOLFIRI ± bevacizumab arm and 15.7 months in the veliparib plus FOLFIRI ± bevacizumab arm, based on a minimum of 70 PFS events, the expected 95% confidence interval for the estimated hazard ratio would be approximately 0.37–0.96.

One cycle of therapy was 14 days, from Day −2 through Day 12. Dosing of oral veliparib/placebo began 2 days prior to the start of FOLFIRI and continued twice a day (BID) for 7 consecutive days. At the discretion of the investigator, bevacizumab (5 mg/kg) was administered intravenously immediately preceding FOLFIRI. Patients randomised to the veliparib arm received modified FOLFIRI as irinotecan 180 mg/m^2^ (90-min infusion ± 30 min); leucovorin 400 mg/m^2^ (90-min infusion ± 30 min); saline bolus (up to 15 min infusion) immediately followed by 5-fluorouracil (5-FU) 2400 mg/m^2^ (46 h continuous infusion ± 4 h) starting on Day 1 of each cycle. Patients randomised to the placebo arm received standard FOLFIRI as irinotecan 180 mg/m^2^ (90-min infusion ± 30 min); leucovorin 400 mg/m^2^ (90-min infusion ± 30 min); 5-FU bolus 400 mg/m^2^ (up to 15-min infusion) immediately followed by 5-FU 2400 mg/m^2^ (46 h continuous infusion ± 4 h) on Day 1 of each cycle. FOLFIRI was only given after veliparib/placebo dosing on Cycle Day −2 and Day −1 was confirmed. Patients continued to follow the dosing schedule for therapy until disease progression or other criteria for discontinuation were met.

Screening procedures and baseline radiographic tumour assessments were performed within 28 days prior to the first dose of veliparib/placebo on Cycle 1 Day −2. Study visits were conducted on Day 1 and Day 8 of the first and second cycle, then on Day 1 of each subsequent cycle. Post-baseline tumour assessment was conducted every 8 weeks from Cycle 1 Day 1 (prior to the start of a new cycle) until radiographic progression. Patients with controlled disease (complete response [CR], partial response [PR], or stable disease [SD]) and with tolerable side effects continued to receive protocol therapy until disease progression. All patients had one follow-up visit approximately 30 days after the last dose of therapy.

### Safety and tolerability

Safety analysis was conducted for all patients who received at least one dose of veliparib. Treatment-emergent adverse events and lab abnormalities were reported according to the National Cancer Institute Common Terminology Criteria for Adverse Events (CTCAE) version 4.0.

### Statistical analyses

The primary endpoint was PFS, defined as the number of days from the date the patient was randomised to the date the patient experienced a disease progression event or to the date of death if disease progression was not reached. The distribution of PFS was estimated for each treatment group using Kaplan–Meier methodology. mPFS time was estimated and 95% confidence interval for the estimated mPFS time is presented for each treatment group. The Cox Proportional Hazard Model was used to estimate the hazard ratio and 95% confidence interval comparing the two treatment groups, stratified by planned bevacizumab use (planned use versus no planned use).

For overall survival (OS) analysis, time to death was defined as the number of days from the day the patient was randomised to the date of the patient’s death. All events of death were included, regardless of whether the event occurred while the patient was still taking study drug, or after the patient discontinued study drug. If a patient has not died, then the data were censored at the date when the patient was last known to be alive. The distribution of OS was estimated for each treatment group using Kaplan–Meier methodology. Median survival time was estimated and 95% confidence interval for the estimated median survival time is presented for each treatment group. The Cox Proportional Hazard Model was used to estimate the hazard ratio and 95% confidence interval comparing the two treatment groups, stratified by planned bevacizumab use (planned use versus no planned use).

Objective response rate (CR and PR) was defined as the proportion of patients with a complete or partial objective response based on RECIST, version 1.1. Objective response rate was estimated and compared between the two treatment groups using a Cochran–Mantel–Haenszel test, stratified by the planned bevacizumab use. A 95% confidence interval was constructed for the estimated proportions.

The primary analysis data cut-off was 31 December 2016. This clinical trial was completed and the final data cut-off for analysis was 31 October 2017.

## Results

### Patient characteristics

As of 31 October 2017, 130 patients were randomised to receive either veliparib (*N* = 65) or placebo (*N* = 65). Patient demographics and clinical characteristics are shown in Table 1. Median age was 59 years in the veliparib group vs 64 years in the placebo group (*p* = 0.016 one-way ANOVA test). Other demographic characteristics were balanced across treatment groups. A majority of patients were White and about half of patients received bevacizumab.

**Table 1:** Patient demographic and baseline clinical characteristics

*n*, %	Veliparib + FOLFIRI ± Bevacizumab (*N* = 65)	Placebo + FOLFIRI ± Bevacizumab (*N* = 65)	*P*-value
Age, years, median (range)	59 (26–84)	64 (43–84)	0.016^a^
**Age**			0.048^b^
**<**65 years	45 (69%)	33 (51%)	
≥65 years	20 (31%)	32 (49%)	
**Gender**			–
Male	44 (68%)	40 (62%)	
Female	21 (32%)	25 (38%)	
**Race**			–
White	62 (95%)	64 (98%)	
Black	3 (5%)	1 (2%)	
**Ethnicity**			–
Hispanic or Latino	2 (3%)	7 (11%)	
Not Hispanic or Latino	63 (97%)	58 (89%)	
**World region**			–
North America	8 (12%)	9 (14%)	
Rest of world	57 (88%)	56 (86%)	
**Bevacizumab use (actual)**			–
Received	31 (48%)	32 (49%)	
Not received	34 (52%)	33 (51%)	
**ECOG Performance Status**			–
0	26 (40%)	24 (37%)	
>0	39 (60%)	41 (63%)	
**Smoking status**			–
Current	10 (15%)	10 (15%)	
Former	16 (25%)	26 (40%)	
Never	37 (57%)	29 (45%)	
Unknown	2 (3%)	0	
**Number of metastatic sites**			–
1	44 (68%)	42 (65%)	
2	14 (22%)	14 (22%)	
3	4 (6%)	4 (6%)	
4	3 (5%)	5 (8%)	
**Location of metastases**			–
Lung	24 (37%)	24 (37%)	
Non-lung	41 (63%)	41 (63%)	
**Tumour resection history**			–
Yes	36 (55%)	39 (60%)	

^a^ *P*-value for differences between treatment groups from one-way ANOVA test

^b^ *P*-value for differences between treatment groups from Fisher’s Exact Test.

Only *P*-values<0.05 are presented

The mean number of cycles of exposure to veliparib, fluorouracil, irinotecan, and bevacizumab were similar across both veliparib and placebo treatment groups (Supplemental Table S1) with mean exposure (± SD) of 16 ± 13 and 19 ± 13 cycles in veliparib and placebo arms, respectively. Fluorouracil bolus exposure was 7 ± 10 and 10 ± 11 cycles; fluorouracil infusion exposure was 16 ± 12 and 18 ± 13 cycles. Irinotecan exposure was 16 ± 12 and 18 ± 13 cycles. Bevacizumab exposure was 15 ± 12 and 16 ± 12 cycles.

### Efficacy

**Fig. 1:**
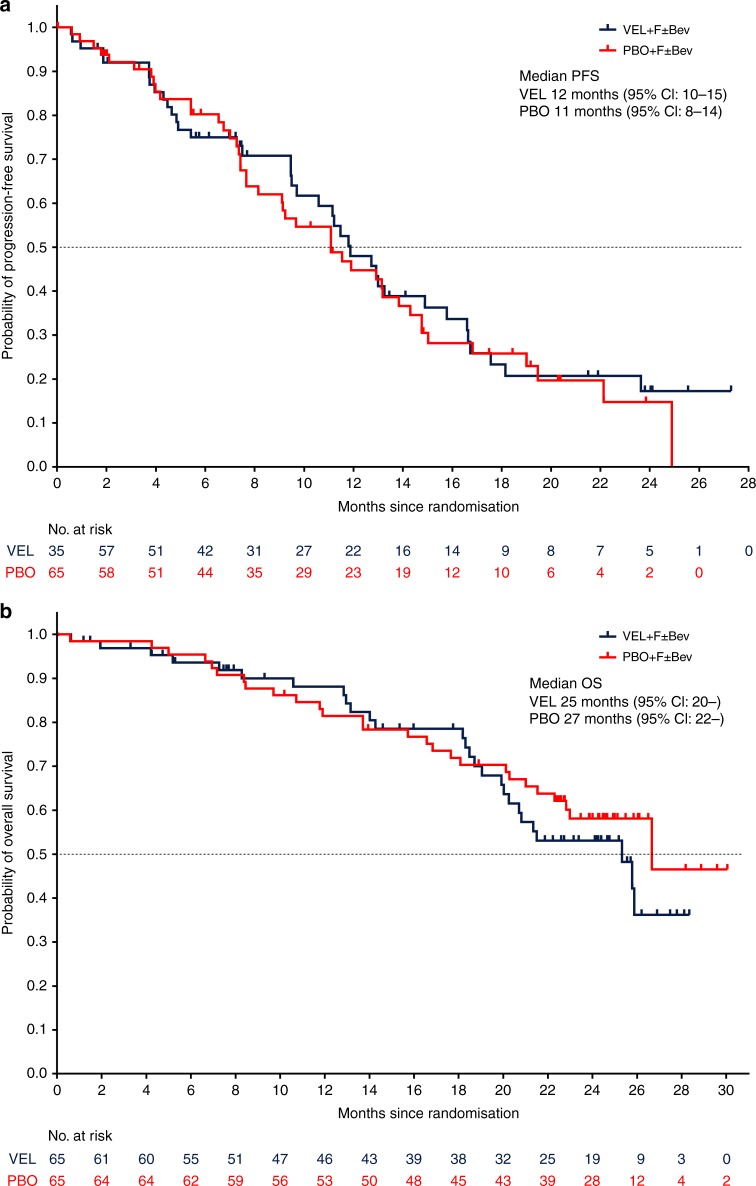
**a** Progression-free survival at final analysis and **b** overall survival at final analysis. CI confidence interval, PBO placebo + FOLFIRI ± bevacizumab, VEL veliparib + FOLFIRI ± bevacizumab

The primary endpoint of mPFS was 12 vs 11 months (veliparib vs placebo) [HR = 0.94 (95% CI: 0.60, 1.48)] at primary analysis (31 December 2016). At final analysis (31 October 2017), mPFS was 12 vs 11 months (veliparib vs placebo) [HR = 0.91 (95% CI: 0.60, 1.41)] (Fig.1a). Median OS (mOS) was 25 vs 27 months (veliparib vs placebo) [HR = 1.26 (95% CI: 0.74, 2.16)] at final analysis. There were 27 death events in each arm (veliparib vs placebo) (Fig.1b). Using the reverse Kaplan–Meier method, the median follow-up time for the study is 23.8 months (95% CI: 22.6, 24.3).

Veliparib added to FOLFIRI demonstrated similar mPFS and mOS as placebo did, regardless of bevacizumab use (Supplemental Figures S1, S2). Patients with planned bevacizumab use had mPFS of 13 vs 14 months (veliparib vs placebo, *n* = 32 each group) and mOS of 26 vs 27 months, respectively. Patients with no planned bevacizumab use had mPFS of 10 vs 8 months (veliparib vs placebo, *n* = 33 each group) and mOS of 25 months for the veliparib group.

**Fig. 2:**
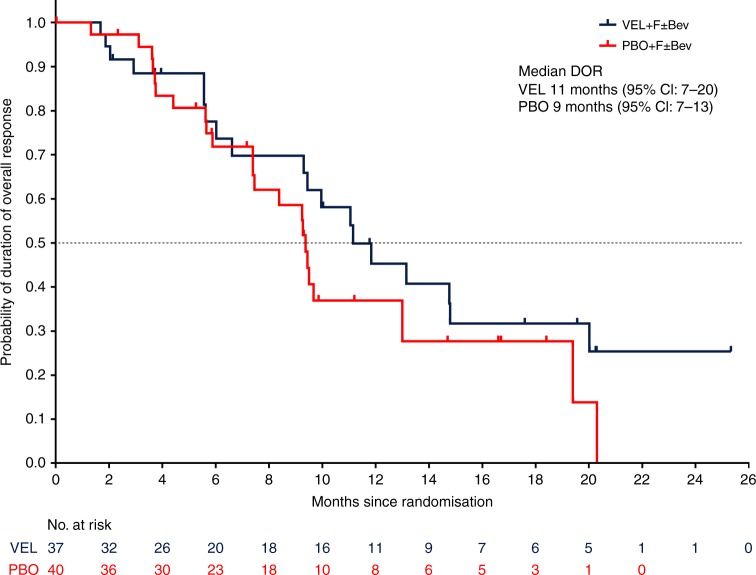
Duration of overall response at final analysis. CI confidence interval, PBO placebo + FOLFIRI ± bevacizumab, VEL veliparib + FOLFIRI ± bevacizumab

Objective response rate was 57% for the veliparib treatment group and 62% for the placebo group (Table 2). The median duration of overall response was 11 vs 9 months for veliparib vs placebo, respectively [HR = 0.73 (95% CI: 0.38, 1.40)] at primary analysis. At final analysis, duration of overall response was numerically longer for patients treated with veliparib compared to placebo with 11 vs 9 months for veliparib vs placebo [HR = 0.75 (95% CI: 0.41, 1.40)] (Fig. 2).

**Table 2:** Objective response rates

*n*, %	Veliparib + FOLFIRI ± Bevacizumab (*N* = 65)	Placebo + FOLFIRI ± Bevacizumab (*N* = 65)
ORR [95% CI]	37 (57%) [44.0, 69.2]	40 (62%) [48.6, 73.3]
**Best response**		
Complete response	2 (3%)	2 (3%)
Partial response	35 (54%)	38 (59%)
Stable disease	20 (31%)	19 (29%)
Disease progression	3 (5%)	4 (6%)
Incomplete data	5 (8%)	2 (3%)

*ORR* objective response rate (CR + PR) measured by RECIST

### Safety

Overall, 123 of the 130 patients (95%) in the “as treated patients” analysis set experienced at least one adverse event during the study. The most common adverse events occurring in ≥20% of patients in either treatment group are presented in Supplemental Table S2A. Common adverse events that had a statistically significant difference (*p* < 0.05) in frequency between veliparib and placebo treatment groups were anaemia (39% vs 19%, *p* = 0.019) and neutropenia (66% vs 37%, *p* = 0.001). Common adverse events that had a significantly higher frequency in the placebo treatment group were abdominal pain (19% vs 37%, *p* = 0.030), constipation (15% vs 32%, *p* = 0.039), mucosal inflammation (6% vs 22%, *p* = 0.020), and epistaxis (9% vs 26%, *p* = 0.020), as compared to the veliparib treatment group.

Common grade 3 or 4 adverse events occurring in ≥5% of patients for veliparib vs placebo treatment groups included neutropenia (59% vs 22%, *p* < 0.001), diarrhoea (17% vs 12%) and asthenia (9% vs 3%), but only neutropenia was significantly different between groups (Supplemental Table S2B). Serious adverse events (for veliparib vs placebo treatment groups) included diarrhoea (14% vs 3%) and febrile neutropenia (5% vs 5%); however, none of the serious adverse events were significantly different (*p* < 0.05) between treatment groups (Supplemental Table S2C). All grade haematopoietic cytopenias were more common with veliparib than placebo (79% vs 52%, *p* = 0.003).

Fourteen percent of patients in the veliparib arm and 15% in the placebo arm prematurely discontinued treatment due to adverse events. Two treatment-emergent grade 5 adverse events occurred in each treatment arm, due to cardiac arrest (two patients in placebo arm), cerebrovascular accident (one patient in veliparib arm) or acute kidney injury (one patient in veliparib arm).

Adverse events of special interest in the veliparib treatment group included haematologic cytopenias (anaemia *p* = 0.019, and neutropenia *p* = 0.001), which were reported at a higher frequency in the veliparib treatment group compared to the placebo treatment group, with a statistically significant difference between the two groups. Adverse events of special interest considered serious that occurred with higher frequency in the veliparib treatment group were nausea, vomiting, haematopoietic cytopenias, haematopoietic leukopenia, neutropenia, and lymphopenia (no statistical difference observed between-treatment groups) (Supplemental Table S3).

## Discussion

This study was a randomised, blinded, placebo-controlled trial conducted at multiple centres around the globe to evaluate the efficacy and tolerability of the addition of veliparib to the standard-of-care regimen, FOLFIRI. Bevacizumab was allowed at the discretion of the investigator. The primary efficacy analysis was a comparison of PFS between veliparib and the placebo treatment groups. mPFS was 361 days (12 months) and 337 days (11 months), respectively, and the study did not show significant difference between the two treatment groups. There were no significant differences in the secondary or tertiary endpoints of OS, objective response rate, or duration of response. However, the duration of response was numerically longer in patients who achieved a response to veliparib treatment compared to placebo treatment, although this difference was not statistically significant. Nine patients in the placebo arm and eight patients in the veliparib arm had surgery for curative intent of metastatic disease and were expected to have similar effect on efficacy analysis. Reduction in tumour size after surgery was not counted in tumour response per RECIST 1.1.

Treatment effect in subgroups was analysed. Addition of veliparib to FOLFIRI demonstrated similar efficacy as FOLFIRI regardless if bevacizumab was used or not. A trend to favour the veliparib treatment group in duration of response and PFS was observed in the ECOG = 0 subgroup and age≥65 years subgroup, although, none of these met statistical significance.

Significantly more patients treated with veliparib experienced all-grade adverse events of blood and lymphatic disorders compared to placebo-treated patients. Neutropenia was the only grade ≥3 adverse event that occurred with a higher frequency in the veliparib treatment group. Anaemia and neutropenia were the adverse events of special interest that occurred at higher frequency in the veliparib group. Serious adverse events were similar between treatment groups. Similar numbers of patients were discontinued, experienced dose delay, reduction and/or interruption of study treatment in both treatment groups. The most common adverse event that led to veliparib dose interruption or reduction was neutropenia.

Overall, the main class of adverse events that occurred at a higher frequency in veliparib group was haematologic toxicities. However, overall treatment tolerability was similar between the treatment groups, as represented by similar frequencies of serious adverse events and adverse events leading to treatment discontinuation between the two groups. The safety profile is consistent with the mechanism of action by veliparib where inhibition of PARP impedes the repair of DNA damage caused by FOLFIRI, and thus potentates the cytotoxicity by FOLFIRI.

Pharmacodynamic evidence was not obtained in this study nor in our phase I study^23^, and the benefit of addition of veliparib to cytotoxic chemotherapy has thus far mainly been shown in preclinical models. The effect of veliparib on the repair of DNA damage induced by FOLFIRI or irinotecan is thought to be mediated by PARP inhibition.^24^ Pommier and co-workers^25,26,20^ have demonstrated that PARP1 is a key component driving DNA repair from topoisomerase I-induced damage, by coupling to the repair enzyme tyrosyl-DNA-phosphodiesterase 1 (TDP1).

There are multiple hypotheses why addition of veliparib did not demonstrate enhanced efficacy in the current study. The dosing and, thus, the efficacy in the veliparib arm may be limited by tolerability. FOLFIRI itself may reach the maximal tolerated dose. Addition of veliparib potentiates the effect of FOLFIRI as supported by the increased incidence of haematological toxicities (Table 3). As the result, the average dosing cycles in the veliparib arm were shorter for all the drug components (Supplementary Table S1). In other words, the higher effect in each cycle is balanced out by the shorter treatment duration in the veliparib arm and the final efficacy was the same as the control arm. Another potential explanation is the dose of veliparib is not optimal to achieve the best balance between enhanced toxicity and efficacy.

**Table 3:** Overview of treatment-emergent adverse events

*n*, %	Veliparib + FOLFIRI ± Bevacizumab (*N* = 65)	Placebo + FOLFIRI ± Bevacizumab (*N* = 65)	*P*-value^a^
All grade AE	62 (95%)	61 (94%)	–
All grade AE related to veliparib	43 (66%)	44 (68%)	–
Grade 3 or 4 AE	53 (82%)	51 (79%)	–
Grade 3 or 4 AE related to veliparib	23 (35%)	21 (32%)	–
SAE	30 (46%)	33 (51%)	–
SAE related to veliparib	8 (12%)	8 (12%)	–
AE leading to veliparib discontinuation^b^	12 (19%)	14 (22%)	–
AE leading to veliparib reduction or interruption	44 (68%)	45 (69%)	–
All grade haematopoietic cytopenias	51 (79%)	34 (52%)	0.003
Haematopoietic erythropenias	25 (39%)	12 (19%)	0.019
Haematopoietic leukopenias	47 (72%)	28 (43%)	0.001
Any neutropenia and lymphopenia	46 (71%)	28 (43%)	0.002
Fatal AE	2 (3%)	2 (3%)	–
Deaths^c^	27 (42%)	27 (42%)	–

*AE* adverse event

^a^ *P*-value for difference between treatment groups from Fisher’s Exact Test. Only *P*-values <0.05 are presented

^b^ Includes adverse events related to progression and not related to progression

^c^ Includes all treatment-emergent deaths and deaths that occurred >30 days after last dose

Other PARP inhibitors have been combined with irinotecan in colorectal cancer, but with limited success as well. Olaparib combined with the active metabolite of irinotecan, SN-38, showed synergistic effect in colon cancer cells, by potentiating the double-strand DNA breaks induced by SN-38.^27^ However, a phase 1 study of olaparib with irinotecan in patients with colorectal cancer reported high toxicity concerns and no antitumour efficacy.^28^

The current study has some limitations. A notable difference between the two treatment arms was that the median age of patients was significantly younger for veliparib vs placebo arms (59 vs 64 years, respectively; *p* = 0.016), with more patients aged under 65 years in the veliparib arm than patients over 65 years. The ECOG performance status was not significantly different between treatment arms, with the ECOG = 0 group comprising 40% versus 37% in the veliparib and placebo arms, respectively. Other limitations of our study included the small sample size. While the 5-FU bolus was not expected to be important for efficacy by many physicians and skipping bolus in the veliparib arm was mainly for safety considerations, this is one limitation of the trial.

In conclusion, analysis of survival and response rate of this phase 2 study showed that addition of veliparib to FOLFIRI did not significantly improve overall treatment outcome. Consistent with the mechanism of action, veliparib added to FOLFIRI regimen significantly increased haematologic adverse events. Safety data were in alignment with previous combination therapy studies using veliparib, and no new safety concerns were observed.

## Electronic supplementary material

Supplemental Material

## Data Availability

AbbVie is committed to responsible data sharing regarding the clinical trials we sponsor. Access is provided to anonymised, patient and trial-level data (analysis data sets), as well as other information (e.g., protocols and Clinical Study Reports) from AbbVie-sponsored Phase II–IV global interventional clinical trials conducted in patients (completed as of May 2004, for products and indications approved in either the United States or the European Union), as long as the trials are not part of an ongoing or planned regulatory submission. This includes requests for clinical trial data for unlicensed products and indications. Access to this clinical trial data can be requested by any qualified researchers who engage in rigorous, independent scientific research, and will be provided following review and approval of a research proposal and Statistical Analysis Plan (SAP) and execution of a Data Sharing Agreement (DSA). Data requests can be submitted at any time and the data will be accessible for 12 months, with possible extensions considered. For more information on the process, or to submit a request, visit the following link: https://www.abbvie.com/our-science/clinical-trials/clinical-trials-data-and-information-sharing/data-and-information-sharing-with-qualified-researchers.html.
