# Correction: A phase 2 randomised study of veliparib plus FOLFIRI±bevacizumab versus placebo plus FOLFIRI±bevacizumab in metastatic colorectal cancer

**DOI:** 10.1038/s41416-019-0528-0

**Published:** 2019-07-26

**Authors:** Vera Gorbunova, J. Thaddeus Beck, Ralf-Dieter Hofheinz, Pilar Garcia-Alfonso, Marina Nechaeva, Antonio Cubillo Gracian, Laszlo Mangel, Elena Elez Fernandez, Dustin A. Deming, Ramesh K. Ramanathan, Alison H. Torres, Danielle Sullivan, Yan Luo, Jordan D. Berlin

**Affiliations:** 1 N.N. Blokhin Russian Cancer Research Center, Moscow, Russia; 2 Highlands Oncology, Rogers/Fayetteville, AR USA; 3 Interdisciplinary Tumor Center, University Hospital Mannheim, University of Heidelberg, Heidelberg, Germany; 4 Hospital General Universitario Gregorio Marañón, Madrid, Spain; 5 Arkhangelsk Clinical Oncology Center, Arkhangelsk, Russia; 6 Centro Integral Oncológico Clara Campal Hospital Universitario Madrid Sanchinarro, Madrid, Spain, and Departamento de Ciencias Médicas Clínicas, Universidad CEU San Pablo, Madrid, Spain; 7 Pecsi Tudomanyegyetem Klinikai Kozpont, Onkoterapias Intezet, Pécs, Hungary; 8 Vall d’Hebron University Hospital, Barcelona, Spain; 9 University of Wisconsin, Madison, WI USA; 10 Mayo Clinic, Scottsdale, AZ USA; 11 AbbVie Inc., North Chicago, IL USA; 12 Vanderbilt-Ingram Cancer Center, Nashville, TN USA

**Keywords:** Colorectal cancer, Phase II trials, Chemotherapy, Colorectal cancer

**Correction to**: *British Journal of Cancer* (2019) **120**, 183–189; 10.1038/s41416-018-0343-z; published online 11 December 2018

The original version of this article contained an error in Fig. 1a. The number of patients at risk listed in the Veliparib arm of Fig. 1a should have read “65” instead of “35”. The correct figure is below.

**Fig. 1:**
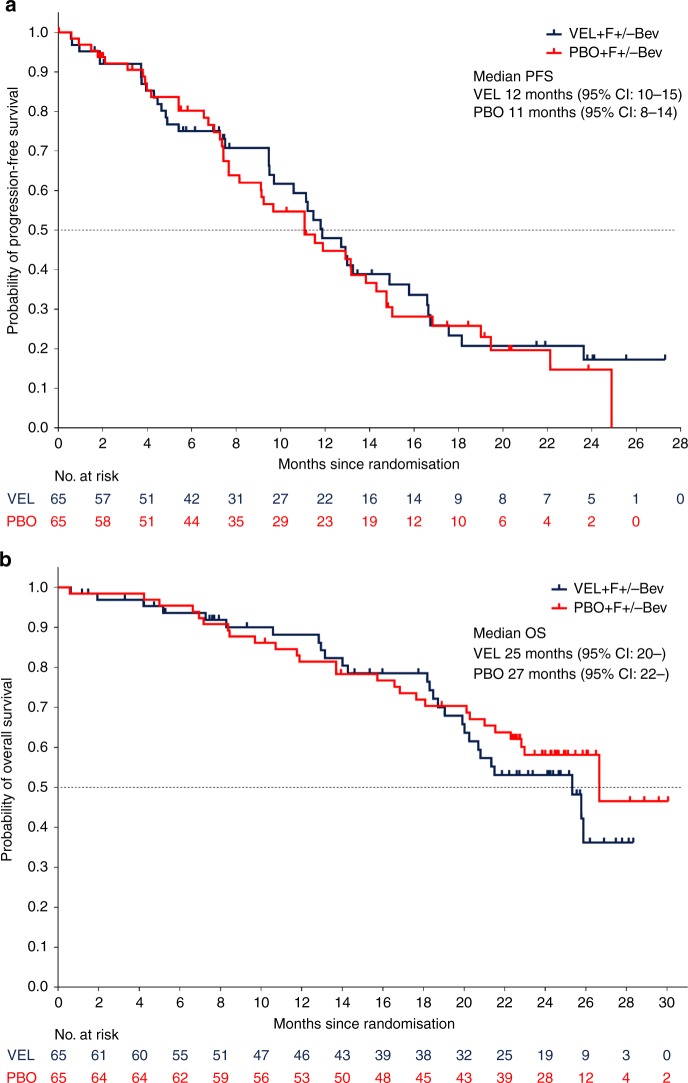
**a** Progression-free survival at final analysis and **b** overall survival at final analysis. CI confidence interval, PBO placebo + FOLFIRI ± bevacizumab, VEL veliparib + FOLFIRI ± bevacizumab

