# Supplementary material for: A phase 2 randomised study of veliparib plus FOLFIRI±bevacizumab versus placebo plus FOLFIRI±bevacizumab in metastatic colorectal cancer
Source: Br J Cancer. 2018 Dec 11;120(2):183–9. doi: 10.1038/s41416-018-0343-z (PMC6342906; doi:10.1038/s41416-018-0343-z)

**Supplementary Materials for the manuscript:**

“A phase 2 randomised study of veliparib plus FOLFIRI ± bevacizumab versus placebo plus FOLFIRI ± bevacizumab in metastatic colorectal cancer” by Vera Gorbunova *et al.*

**Supplementary Table S1. Summary of patient exposure to study treatment**

| **Mean no. of cycles for each drug component (SD), [Range]** | **Veliparib + FOLFIRI**  **± Bevacizumab** | **Placebo + FOLFIRI**  **± Bevacizumab** | **P-value^a^** |
| --- | --- | --- | --- |
|  | **N=65** | **N=65** |  |
| **Veliparib** | 16 (13)  [1-49] | 19 (13)  [1-53] | 0.295 |
| **Fluorouracil bolus** | 7 (10)  [1-48] | 10 (11)  [1-52] | 0.145 |
| **Fluorouracil infusion** | 16 (12)  [1-49] | 18 (13)  [1-52] | 0.448 |
| **Irinotecan** | 16 (12)  [1-48] | 18 (13)  [1-52] | 0.369 |
|  | **N=31** | **N=32** |  |
| **Bevacizumab** | 15 (12)  [1-48] | 16 (12)  [1-45] | 0.582 |

^a^ P-value for differences in mean between two treatment groups is from one-way ANOVA.

**Supplementary Table S2. (A) All grade adverse events in ≥20% of patients in either treatment group, (B) grade 3/4 adverse events in ≥5% of patients in either treatment group, and (C) serious adverse events occurring in at least two patients overall**

**A.**

| **Adverse event, n (%)** | **Veliparib + FOLFIRI**  **± Bevacizumab**  **(N=65)** | **Placebo + FOLFIRI**  **± Bevacizumab**  **(N=65)** | **P-value^a^** |
| --- | --- | --- | --- |
| Neutropenia | 43 (66%) | 24 (37%) | 0.001 |
| Diarrhea | 38 (59%) | 37 (57%) |  |
| Nausea | 36 (55%) | 40 (62%) |  |
| Vomiting | 28 (43%) | 26 (40%) |  |
| Fatigue | 25 (39%) | 24 (37%) |  |
| Alopecia | 25 (39%) | 18 (28%) |  |
| Anemia | 25 (39%) | 12 (19%) | 0.019 |
| Asthenia | 16 (25%) | 14 (22%) |  |
| Decreased appetite | 15 (23%) | 19 (29%) |  |
| Stomatitis | 15 (23%) | 10 (15%) |  |
| Abdominal pain | 12 (19%) | 24 (37%) | 0.030 |
| Constipation | 10 (15%) | 21 (32%) | 0.039 |
| Epistaxis | 6 (9%) | 17 (26%) | 0.020 |
| Mucosal inflammation | 4 (6%) | 14 (22%) | 0.020 |

**B.**

| **Grade 3 or 4 adverse event, n (%)** | **Veliparib + FOLFIRI**  **± Bevacizumab**  **(N=65)** | **Placebo + FOLFIRI**  **± Bevacizumab**  **(N=65)** | **P-value^a^** |
| --- | --- | --- | --- |
| Neutropenia | 38 (59%) | 14 (22%) | <0.001 |
| Diarrhea | 11 (17%) | 8 (12%) |  |
| Asthenia | 6 (9%) | 2 (3%) |  |
| Febrile neutropenia | 5 (8%) | 3 (5%) |  |
| Anemia | 5 (8%) | 2 (3%) |  |
| Leukopenia | 5 (8%) | 2 (3%) |  |
| Nausea | 5 (8%) | 2 (3%) |  |
| Dehydration | 5 (8%) | 2 (3%) |  |
| Fatigue | 4 (6%) | 4 (6%) |  |
| Hypokalemia | 4 (6%) | 2 (3%) |  |
| Pulmonary embolism | 2 (3%) | 6 (9%) |  |
| Hyperglycemia | 2 (3%) | 5 (8%) |  |
| Hypertension | 3 (5%) | 5 (8%) |  |

**C.**

| **Adverse event, n (%)** | **Veliparib + FOLFIRI**  **± Bevacizumab**  **(N=65)** | **Placebo + FOLFIRI**  **± Bevacizumab**  **(N=65)** | **P-value^a^** |
| --- | --- | --- | --- |
| Diarrhea | 9 (14%) | 2 (3%) | - |
| Febrile neutropenia | 3 (5%) | 3 (5%) | - |
| Intestinal obstruction | 1 (2%) | 3 (5%) | - |
| Large intestinal obstruction | 2 (3%) | 1 (2%) | - |
| Neutropenia | 2 (3%) | 1 (2%) | - |
| Pneumonia | 2 (3%) | 1 (2%) | - |
| Abdominal abscess | 1 (2%) | 1 (2%) | - |
| Abdominal pain | 0 | 2 (3%) | - |
| Acute kidney injury | 1 (2%) | 1 (2%) | - |
| Bile duct stenosis | 2 (3%) | 0 | - |
| Cadiac arrest | 0 | 2 (3%) | - |
| Cerebrovascular accident | 1 (2%) | 1 (2%) | - |
| Deyhdration | 1 (2%) | 1 (2%) | - |
| Pulmonary embolism | 0 | 2 (3%) | - |
| Dyspnea | 0 | 2 (3%) | - |
| Malignant neoplasm progression | 0 | 2 (3%) | - |
| Pyrexia | 2 (3%) | 0 | - |
| Vomiting | 2 (3%) | 0 | - |

^a^ P-value for comparisons between treatment groups using Fisher's Exact Test.
Part A-B: Only P-values ≤0.1 are presented. Part C: Only P-values <0.05 are presented.

**Supplementary Table S3. Adverse events of special interest considered serious**

| **Adverse event, n (%)** | **Veliparib + FOLFIRI**  **± Bevacizumab**  **(N=65)** | **Placebo + FOLFIRI**  **± Bevacizumab**  **(N=65)** |
| --- | --- | --- |
| Nausea | 1 (2%) | 0 |
| Vomiting | 2 (3%) | 0 |
| Seizures | 0 | 0 |
| Febrile neutropenia | 3 (5%) | 3 (5%) |
| Neutropenia | 2 (3%) | 1 (2%) |
| Hematopoietic erythropenia | 0 | 0 |
| Hematopoietic thrombocytopenia | 0 | 0 |
| Fertility disorder | 0 | 0 |
| Malignant neoplasm progression | 0 | 2 (3%) |
| Metastases to central nervous system | 0 | 1 (2%) |
| Myelodysplastic syndrome | 0 | 0 |

**Supplementary Figure S1. Progression-free survival by (A) planned bevacizumab use and (B) not planned bevacizumab use**

**A.**


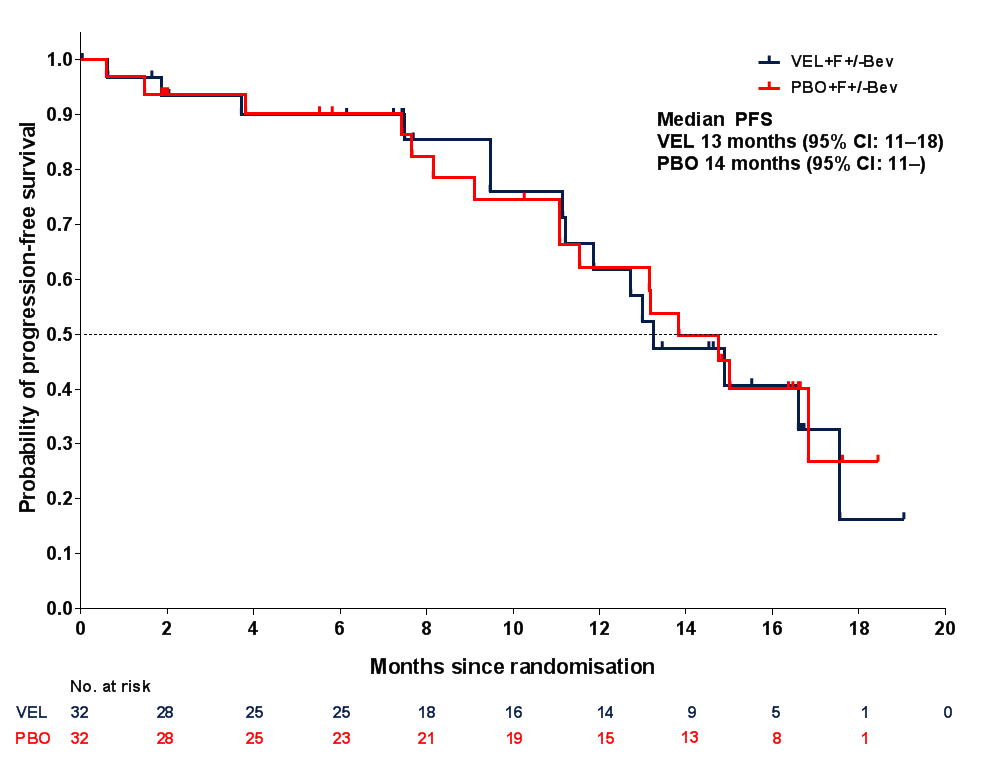


**B.**


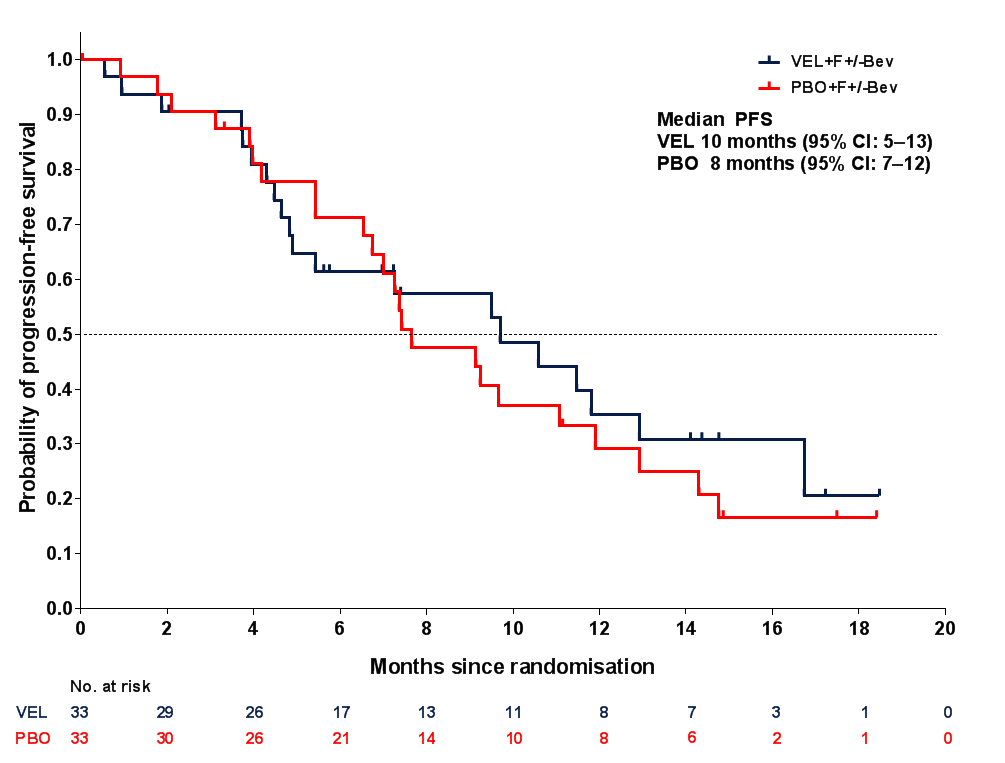


**Supplementary Figure S2. Overall survival by (A) planned bevacizumab use and (B) not planned bevacizumab use**

**A.**


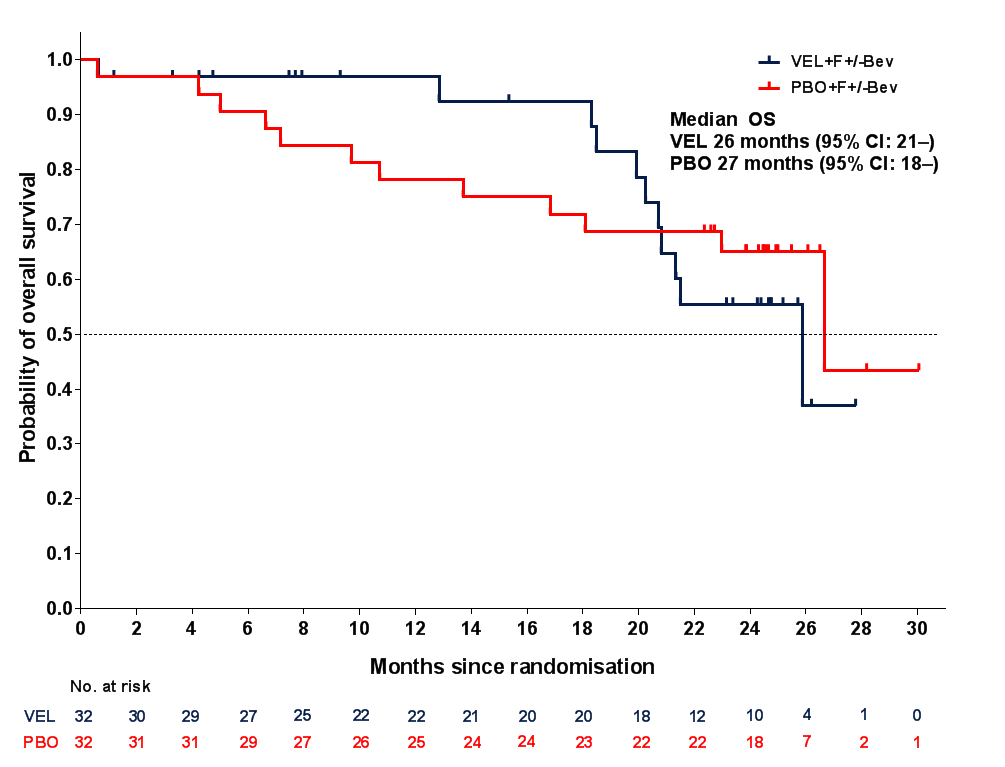


**B.**


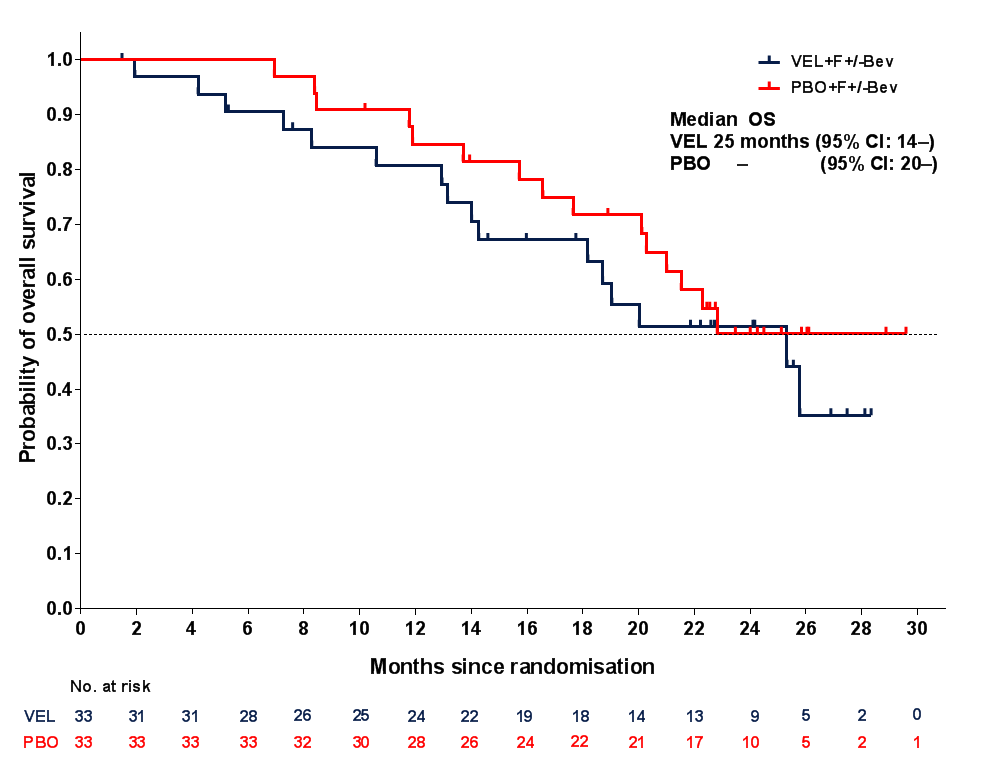

Supplement: Supplementary file 1 — Supplemental Material [file 41416_2018_343_MOESM1_ESM.docx]
